# Characterizing occupational radon exposure greater than 100 Bq/m^3^ in a highly exposed country

**DOI:** 10.1038/s41598-022-25547-x

**Published:** 2022-12-09

**Authors:** A. Brobbey, E. Rydz, S. Fenton, P. A. Demers, C. B. Ge, C. E. Peters

**Affiliations:** 1 CAREX Canada, School of Population and Public Health, University of British Columbia, Vancouver, BC Canada; 2 Department of Oncology, Cumming School of Medicine, University of Calgary, Calgary, AB Canada; 3 Occupational Cancer Research Centre, Ontario Health, Toronto, ON Canada; 4 Dalla Lana School of Public Health, University of Toronto, Toronto, ON Canada; 5 TNO, The Hague, The Netherlands; 6 BCCDC, Vancouver, BC Canada; 7 BC Cancer, Vancouver, BC Canada

**Keywords:** Risk factors, Cancer prevention

## Abstract

Radon is an established lung carcinogen concentrating in indoor environments with importance for many workers worldwide. However, a systematic assessment of radon levels faced by all workers, not just those with direct uranium or radon exposure, has not previously been completed. The objective of this study was to estimate the prevalence of workers exposed to radon, and the level of exposure (> 100–200 Bq/m^3^, 200–400 Bq/m^3^, 400–800 Bq/m^3^, and > 800 Bq/m^3^) in a highly exposed country (Canada). Exposures among underground workers were assessed using the CAREX Canada approach. Radon concentrations in indoor workplaces, obtained from two Canadian surveys, were modelled using lognormal distributions. Distributions were then applied to the susceptible indoor worker population to yield the number of exposed workers, by occupation, industry, province, and sex. In total, an estimated 603,000 out of Canada’s 18,268,120 workers are exposed to radon in Canada. An estimated52% of exposed workers are women, even though they comprise only 48% of the labour force. The majority (68%) are exposed at a level of > 100–200 Bq/m^3^. Workers are primarily exposed in educational services, professional, scientific and technical services, and health care and social assistance, but workers in mining, quarrying, and oil and gas extraction have the largest number of exposed workers at high levels (> 800 Bq/m^3^). Overall, a significant number of workers are exposed to radon, many of whom are not adequately protected by existing guidelines. Radon surveys across multiple industries and occupations are needed to better characterize occupational exposure. These results can be used to identify exposed workers, and to support lung cancer prevention programs within these groups.

## Introduction

Cancer is the leading cause of death in Canada^1^. In 2021, an estimated 13% of all diagnosed cancers and an estimated 25.5% of cancer deaths were due to lung cancer, making lung cancer the most commonly diagnosed cancer and the leading cause of cancer death in Canada, which is similar to rates in North America and Europe^2^. Lung cancer is also one of the most costly cancers, costing the Canadian publicly funded healthcare system an estimated $2 billion in 2020 alone^3^. However, an estimated 86% of lung cancer cases are attributable to modifiable risk factors and thus technically preventable^4^.

Radon is an established carcinogen based on clear evidence of excess lung cancer rates in miners and experimental animals exposed to radon^5^. Among smokers, the risk of lung cancer is increased, due to the synergistic effects of radon and smoking^6^. After smoking, radon is the second most common cause of lung cancer among smokers, and the leading cause of lung cancer among non-smokers^7^, with an estimated 3–20% of all lung cancer deaths caused by indoor radon exposure worldwide^8^. Radon is a tasteless, colourless, and odourless radioactive gas that is generated as a progeny from the decay of the naturally occurring 238U in soil, rock and water^7^. Radon is transported along rock fractures and through pore spaces in soil and can be released into the atmosphere or seep into buildings through cracks in the foundation, vents, and other entry points. While outdoor concentrations of radon measured in air are usually diluted and low, levels can concentrate in enclosed spaces with poor ventilation, particularly underground and where radon soil concentrations are high, making this a distinctly anthropogenic problem^7^.

In Canada, the Naturally Occurring Radioactive Materials (NORM) Guidelines aim to protect workers working with or around naturally occurring radioactive materials, as well as workers and the public who are incidentally exposed^9^. When the average radon concentration levels exceed 200 Bq/m^3^, the guidelines recommend that administrative and engineering controls be implemented to reduce levels to below 200 Bq/m^3^, and if radon levels exceed 800 Bq/m^3^, a dose monitoring program should be initiated^9^. Health Canada has also set a guideline of 200 Bq/m^3^ for dwellings, which includes workplaces not regulated by the NORM guideline or by the Canadian Nuclear Safety Commission (e.g., uranium mines)^10^. In contrast, the World Health Organization (WHO) has recommended lowering annual average radon levels in indoor residential spaces to less than 100 Bq/m^3^ based on evidence of increased cancer risk at low levels of exposure^7^; relative lifetime lung cancer risk increases by an estimated 16% for every 100 Bq/m^3^ increase in radon concentration^7,11,12^.

While residential radon levels have been surveyed across Canada (and often found to be among the highest in the world)^13^, systematic assessment of radon levels faced by workers is less complete. Past exposure to radon among underground uranium mine workers (e.g. in the Ontario^14^ and Eldorado uranium mines^15^) was high; however, more recently, levels have been reduced due to the introduction of exposure controls. A recent analysis of uranium mine workers in Canada found an average radon exposure of 111 Bq/m^3^ for all underground workers captured by the National Dose Registry between 2004 and 2013^16^. A survey of federal buildings in Canada was conducted starting in 2007 and by August 2020, 3.6% of surveyed buildings exceeded 200 Bq/m^3^^17^. However, variations by province were observed, with up to 8% of federal buildings exceeding 200 Bq/m^3^ in some provinces, and levels as high as 2500 Bq/m^3^ in some workplaces^18^. Additional sampling has been collected for select workplaces, including schools, and provinces^19–23^, but the extent of workers’ exposures to radon in Canada as a whole is not known. Other workers potentially exposed include other underground miners, who may work in environments rich with uranium-impregnated bedrock, water treatment workers, as processing groundwater can lead to the dissolution of radon, below grade workers, including those working in tunnels and subways, and other indoor workers not previously studied^24^.

CAREX Canada is a national exposure surveillance project that estimates the number of Canadians exposed to known and suspected carcinogens, and where possible, the levels of exposure^25^. The objectives of this study are to estimate the prevalence of workers exposed to radon greater than 100 Bq/m^3^, in alignment with WHO’s guidelines, as well as the level of exposure, by province, occupation, industry, and sex, in 2016.

## Results

In 2016, approximately 18,268,120 Canadians were in the labour force, of whom 48% were women. A total of 603,000 workers were exposed to radon at levels above 100 Bq/m^3^ in Canada. Overall, 68% of those exposed were in the > 100–200 Bq/m^3^ group, 23% were exposed at > 200–400 Bq/m^3^, 5.8% were exposed at > 400–800 Bq/m^3^, and 2.8% of workers were exposed to radon above 800 Bq/m^3^.

### Exposure by province/region

Most provinces and territories followed the same trend, with the majority of workers exposed in the lowest category of exposure (> 100–200 Bq/m^3^), except Saskatchewan and the Yukon where fewer workers fell in this category (~ 40%). Additionally, most of the provinces and territories had very few workers (< 3%) exposed to concentration above 800 Bq/m^3^ except Nunavut (11%), Northwest Territories (9.8%), Saskatchewan (8.4%), Yukon (7.4%) and Newfoundland (6.4%) (Table 1).

**Table 1:** Estimated prevalence of occupational exposure to radon by province/territory and level of exposure.

Province/territories	Exposure level (Bq/m^3^)				Total*
	> 100–200	> 200–400	> 400–800	> 800	
Alberta	79,000 (69%)	28,000 (24%)	6300 (5.4%)	1700 (1.5%)	115,000
	19.2%	19.9%	18.0%	10.2%	
British Columbia	52,000 (69%)	17,000 (23%)	4100 (5.5%)	1700 (2.2%)	76,000
	12.6%	12.6%	11.8%	9.9%	
Manitoba	44,000 (60%)	22,000 (29%)	6300 (8.5%)	1800 (2.4%)	75,000
	10.7%	15.8%	18.2%	10.6%	
New Brunswick	7800 (69%)	2600 (23%)	630 (5.5%)	300 (2.6%)	12,000
	1.9%	1.9%	1.8%	1.8%	
Newfoundland	4900 (64%)	1800 (24%)	490 (6.3%)	490 (6.4%)	77,00
	1.2%	1.3%	1.4%	2.9%	
Nova Scotia	5200 (71%)	1600 (21%)	340 (4.7%)	190 (2.6%)	7300
	1.3%	1.1%	1.0%	1.1%	
Northwest Territories	630 (53%)	320 (27%)	120 (10%)	120 (9.8%)	1200
	0.2%	0.2%	0.3%	0.7%	
Nunavut	260 (62%)	90 (21%)	25 (5.5%)	45 (11.2%)	420
	0.1%	0.1%	0.1%	0.3%	
Ontario	112,000 (77%)	26,000 (18%)	4100 (2.8%)	3600 (2.5%)	147,000
	27%	19.1%	11.7%	21.6%	
Prince Edward Island	520 (89%)	60 (10%)	< 5 (1%)	< 5 (1%)	590
	0.1%	0.0%	0.0%	0.0%	
Quebec	83,000 (77%)	20,000 (18%)	3000 (2.8%)	2200 (2.0%)	108,000
	20.0%	14.2%	8.6%	13.0%	
Saskatchewan	22,000 (41%)	18,000 (33%)	8900 (17%)	4500 (8.4%)	54,000
	5.4%	12.9%	25.7%	26.7%	
Yukon	1200 (41%)	1100 (34%)	490 (17%)	220 (7.4%)	3000
	0.3%	0.7%	1.4%	1.3%	
Total	413,000	138,000	35,000	17,000	603,000

*Number of exposed may not add up to total because of rounding; row percentage in bracket; column percentage under each estimated prevalence.

### Exposure by sex

**Figure 1:**
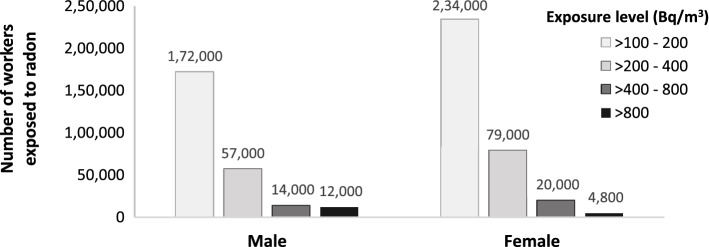
Estimated number of workers exposed to radon by sex and level of exposure.

Of the workers exposed to radon, 57% were female. We found that the proportion of males and females exposed to radon was significantly different in the various exposure groups (Pearson chi^2^(3) = 5500, p value =  < 0.001). As can be seen in Fig. 1, male workers were overrepresented in the highest exposure category (n = 12,000 or 4.7% of males exposed) as compared to female workers (4800 or 1.4% of females exposed).

### Exposure by industry

Table 2 summarizes the estimated number of workers exposed to radon by industry sector (2-digit NAICS code). The sectors with the largest number of workers exposed to radon were educational services (78,000 workers), professional, scientific and technical services (75,000 workers), health care and social assistance (67,000 workers), and public administration (63,000 workers). However, these four industry sectors had relatively fewer exposed workers to > 400 Bq/m^3^ radon concentrations, with most workers primarily exposed to > 100–400 Bq/m^3^ radon concentrations. For all industry sectors listed in Table 2, mining, quarrying, and oil and gas extraction had the largest number of exposed workers in the > 800 Bq/m^3^ radon concentration group (8900 workers). The percentage of exposed population in the mining, quarrying, and oil and gas extraction sector in the > 800 Bq/m^3^ group was 43.6% compared to just 1.5% for educational services, which had the largest population exposed to radon overall.

**Table 2:** Estimated number of workers exposed to radon by level of exposure and North American Industry Classification System (NAICS 2012)—2-digit sector.

NAICS [code]	Exposure level (Bq/m^3^)				Total*
	> 100–200	> 200–400	> 400–800	> 800	
Agriculture, forestry, fishing and hunting [11]	2300 (63.2%)	950 (26.1%)	300 (8.2%)	90 (2.6%)	3700
	0.6%	0.7%	0.9%	0.6%	
Mining, quarrying, and oil and gas extraction [21]	7700 (37.8%)	3000 (14.6%)	830 (4.1%)	8900 (43.6%)	20,000
	1.9%	2.1%	2.4%	52.8%	
Utilities [22]	5300 (67.4%)	1900 (23.8%)	490 (6.3%)	200 (2.5%)	7900
	1.3%	1.3%	1.4%	1.2%	
Construction [23]	24,000 (68.9%)	8200 (23.6%)	2100 (6.1%)	490 (1.4%)	35,000
	5.8%	6.0%	6.1%	2.9%	
Manufacturing [31–33]	21,000 (71.7%)	6600 (22.2%)	1500 (5.1%)	300 (1.0%)	30,000
	5.2%	4.8%	4.3%	1.8%	
Wholesale trade [41]	17,000 (70.3%)	5600 (22.9%)	1400 (5.6%)	300 (1.2%)	25,000
	4.2%	4.1%	3.9%	1.8%	
Retail trade [44–45]	15,000 (70%)	5000 (23.1%)	1200 (5.7%)	260 (1.2%)	22,000
	3.7%	3.6%	3.5%	1.6%	
Transportation and warehousing [48–49]	2500 (63.2%)	870 (22.3%)	230 (6.0%)	330 (8.5%)	3900
	0.6%	0.6%	0.7%	2.0%	
Information and cultural industries [51]	16,000 (70.5%)	5100 (22.7%)	1200 (5.5%)	270 (1.2%)	23,000
	3.9%	3.7%	3.6%	1.6%	
Finance and insurance [52]	33,000 (70.2%)	11,000 (22.9%)	2700 (5.6%)	590 (1.2%)	47,000
	8.0%	7.8%	7.6%	3.5%	
Real estate and rental and leasing [53]	12,000 (70.7%)	4000 (22.7%)	950 (5.4%)	200 (1.1%)	17,000
	3.0%	2.9%	2.7%	1.2%	
Professional, scientific and technical services [54]	53,000 (71%)	17,000 (22.6%)	4000 (5.3%)	830 (1.1%)	75,000
	12.9%	12.3%	11.5%	4.9%	
Management of companies and enterprises [55]	11,00 (68%)	400 (24.0%)	11 0(6.5%)	25 (1.6%)	1700
	0.3%	0.3%	0.3%	0.2%	
Administrative/support, waste management and Remediation service [56]	24,000 (70.8%)	7700 (22.7%)	1800 (5.4%)	380 (1.1%)	34,000
	5.8%	5.6%	5.3%	2.2%	
Educational services [61]	53,000 (68.4%)	19,000 (23.8%)	4900 (6.3%)	1100 (1.5%)	78,000
	12.9%	13.4%	14.1%	6.8%	
Health care and social assistance [62]	46,000 (68.9%)	16,000 (23.7%)	4000 (6.0%)	890 (1.3%)	67,000
	11.1%	11.4%	11.5%	5.3%	
Arts, entertainment, and recreation [71]	7000 (70.1%)	2300 (23.0%)	570 (5.7%)	120 (1.2%)	10,000
	1.7%	1.7%	1.6%	0.7%	
Accommodation and food services [72]	5800 (67.5%)	2100 (24.4%)	560 (6.6%)	130 (1.6%)	8500
	1.4%	1.5%	1.6%	0.8%	
Other services (except public administration) [81]	22,000 (69.2%)	7600 (23.4%)	1900 (6.0%)	440 (1.4%)	32,000
	5.4%	5.5%	5.5%	2.6%	
Public administration [91]	43,000 (68.4%)	15,000 (23.8%)	4000 (6.3%)	950 (1.5%)	63,000
	10.4%	10.8%	11.4%	5.6%	
Total	413,000	138,000	35,000	17,000	603,000

*Number of exposed may not add up to total because of rounding; row percentage in bracket; column percentage under each estimated prevalence.

More specifically, industries with the highest number of exposed workers were elementary and secondary schools (47,000 workers), provincial and territorial public administration (21,000 workers), universities (20,000 workers), and federal protective services (20,000 workers) (Table 3).

**Table 3:** Estimated number of workers exposed to radon by level of exposure and top 10 4-digit Industries (NAICS 2012).

NAICS	Exposure level (Bq/m^3^)				Total*
	> 100–200	> 200–400	> 400–800	> 800	
Elementary and secondary schools [6111]	32,000 (68.3%)	11,000 (23.5%)	3100 (6.6%)	740 (1.6%)	47,000
Provincial and territorial administration [9120]	14,000 (66.7%)	5100 (24.3%)	1500 (7.1%)	380 (1.8%)	21,000
Colleges, universities, and professional schools [6113]	14,000 (69.4%)	4700 (23.3%)	1200 (5.9%)	270 (1.3%)	20,000
Federal protective services [9112]	14,000 (70.5%)	4500 (22.7%)	1100 (5.5%)	250 (1.3%)	19,000
Local, municipal and regional public administration [9130]	12,000 (69.7%)	4000 (23.2%)	990 (5.7%)	230 (1.3%)	17,000
Depository credit intermediation [5221]	12,000 (71.0%)	3800 (22.5%)	900 (5.3%)	190 (1.1%)	17,000
Computer systems design and related services [5415]	11,000 (73.1%)	3200 (21.3%)	710 (4.7%)	130 (0.9%)	15,000
Building equipment contractors [2382]	10,000 (68.2%)	3500 (23.9%)	930 (6.3%)	230 (1.6%)	15,000
Services to buildings and dwellings [5617]	9900 (70.0%)	3300 (23.3%)	790 (5.6%)	160 (1.1%)	14,000
Child day-care services [6244]	9300 (70.6%)	3000 (22.8%)	720 (5.5%)	150 (1.1%)	13,000

*Number of exposed may not add up to total because of rounding; row percentage in bracket.

### Exposure by occupation

Table 4 shows exposure to radon by job title as coded to the broad occupational category level (1-digit NOC). Business, finance, and administrative occupations had the highest prevalence of exposure to radon (197,000 workers). Occupations in natural resources, agriculture and related industries (i.e. the primary industries) had the lowest prevalence of workers exposed to radon. Although these occupations had the lowest number of exposed workers, almost 90% of the exposed population were in the > 800 Bq/m^3^ concentration group, which is the largest group of workers exposed to high levels of radon among all occupations. Health occupations had the least number of exposed workers in the > 800 Bq/m^3^ exposure group. As shown in Table 4, the number of workers exposed in the four radon concentrations decreased with increasing concentrations (100–800 Bq/m^3^) for all occupations except for workers in primary industry, which includes mining.

**Table 4:** Estimated number of workers exposed to radon by level of exposure and National Occupational Classification system (NOC 2016)—1-digit broad occupation.

NOC	Exposure level (Bq/m^3^)				Total*
	> 100–200	> 200–400	> 400–800	> 800	
Management occupations [0]	47,000 (70.1%)	15,000 (23.0%)	3800 (5.7%)	830 (1.2%)	67,000
	11.4%	11.1%	10.9%	4.9%	
Business, finance, and administrative occupations [1]	137,000 (69.4%)	46,000 (23.4%)	12,000 (5.9%)	2600 (1.3%)	197,000
	33.1%	33.2%	33.4%	15.5%	
National and applied sciences and related occupations [2]	40,000 (70.9%)	13,000 (22.6%)	3000 (5.4%)	640 (1.1%)	56,000
	9.6%	9.2%	8.6%	3.8%	
Health occupations [3]	9500 (69.4%)	3200 (23.5%)	800 (5.9%)	170 (1.3%)	14,000
	2.3%	2.3%	2.3%	1.0%	
Occupations in education, law and social, community and government services [4]	79,000 (68.9%)	27,000 (23.6%)	7000 (6.1%)	1600 (1.4%)	115,000
	19.1%	19.5%	20.0%	9.5%	
Occupations in art, culture, recreation and sport [5]	13,000 (71.4%)	4100 (22.4%)	940 (5.2%)	180 (1.0%)	18,000
	3.1%	2.9%	2.7%	1.1%	
Sales and service [6]	59,000 (69.6%)	20,000 (23.3%)	5000 (5.8%)	1100 (1.3%)	85,000
	14.4%	14.3%	14.3%	6.6%	
Trades, transport and equipment operators and related occupations [7]	22,000 (64.0%)	7700 (22.3%)	2100 (5.9%)	2700 (7.8%)	34,000
	5.3%	5.6%	5.8%	15.8%	
Natural resources, agriculture and related occupations [8]	500 (6.6%)	210 (2.7%)	60 (0.8%)	6800 (89.9%)	7600
	0.1%	0.1%	0.2%	40.4%	
Occupations in manufacturing and utilities [9]	6600 (67.9%)	2300 (23.7%)	600 (6.2%)	220 (2.2%)	9700
	1.6%	1.7%	1.7%	1.3%	
Total	413,000	138,000	35,000	17,000	603,000

*Number of exposed may not add up to total because of rounding; row percentage in bracket; column percentage under each estimated prevalence.

When radon exposure was examined by detailed occupation, the groups with the largest number of exposed workers were administrative assistants (30,000 workers), general office support workers (28,000 workers), receptionists (17,000 workers exposed), and elementary school and kindergarten teachers (16,000 workers) (Table 5).

**Table 5:** Estimated number of workers exposed to radon by level of exposure and top 10 4-digit occupations (NOC 2016).

NOC	Exposure level (Bq/m^3^)				Total*
	> 100–200	> 200–400	> 400–800	> 800	
Administrative assistants [1241]	21,000 (69.5%)	7000 (23.2%)	1800 (6.0%)	420 (1.4%)	30,000
General office support workers [1411]	19,000 (68.6%)	6600 (23.8%)	1700 (6.1%)	380 (1.4%)	28,000
Receptionists [1414]	12,000 (69.2%)	4100 (23.6%)	1000 (5.8%)	240 (1.4%)	17,000
Elementary school and kindergarten teachers [4032]	12,000 (70.0%)	4100 (22.8%)	1000 (5.8%)	240 (1.4%)	16,000
Administrative officers [1221]	11,000 (70.6%)	4100 (22.5%)	880 (5.6%)	200 (1.3%)	15,000
Professional occupations in advertising, marketing and public relations [1123]	10,000 (70.2%)	4100 (23.2%)	780 (5.5%)	160 (1.1%)	15,000
Light duty cleaners [6731]	10,000 (69.7%)	4100 (23.0%)	850 (5.9%)	190 (1.3%)	14,000
Other customer and information services representatives [6552]	8800 (70.9%)	4100 (22.6%)	670 (5.4%)	140 (1.1%)	12,000
Janitors, caretakers and building superintendents [6733]	8000 (67.4%)	4100 (24.4%)	780 (6.6%)	190 (1.6%)	12,000
Early childhood educators and assistants [4214]	7000 (70.2%)	4100 (23.1%)	550 (5.5%)	120 (1.2%)	10,000

*Number of exposed may not add up to total because of rounding; row percentage in bracket.

## Discussion

Historically, radon exposure studies focused on underground uranium miners’ high exposure and their increased risk of lung cancer^7,14,16,26–29^, followed by greater public health concerns about the presence of radon in other environments (e.g., residential homes)^30,31^ and the associated risk of lung cancer in the general population^11,26,27,32–36^. Today, miners’ radon exposure is generally lower than it was historically due to enhanced protective measures^24,28^, but compared to other industry sectors, the mining, quarrying, and oil and gas extraction industry sector still has the greatest prevalence of workers exposed to high radon levels of > 800 Bq/m^3^ (8900 of 17,000 total workers exposed, or 54%).

Our estimates demonstrate that most workers exposed to radon (413,000 of 603,000; or 69%) are exposed to radon at the lowest level (> 100–200 Bq/m^3^) across a variety of industry sectors, including professional, scientific and technical services, educational services, health care and social assistance, and business, finance, and administrative occupations. While greater lung cancer risk is associated with increasing level of radon exposure^7^, no safe level of radon exposure exists^37^, and studies have reported positive associations between relatively low indoor radon concentrations and lung cancer risk^8^. CAREX Canada’s exposure estimates for 2006 were previously applied to estimate the burden of lung cancer cases associated with occupational radon exposure in Canada^38–40^. The results showed that while there is a relatively small lung cancer burden associated with occupational radon exposure overall (0.8% of lung cancers were attributable to occupational radon exposure, compared to 17.5% for tobacco smoking, and 0.8% for second-hand tobacco smoke exposure^41^), low level exposures accounted for the majority of the burden^38^. Of the 188 lung cancer cases attributable to occupational radon exposure during the risk exposure period (1961–2001), 139 cases (or 74%) were associated with radon levels less than 200 Bq/m^3^ due to the very large size of the population exposed at this level^38^.

Despite evidence of lung cancer risk associated with low level exposures to radon^8,38^, existing radon guidelines for the workplace remain inadequate. The United States Environmental Protection Agency (US EPA) recommends dwellings be remediated to reduce radon levels if concentrations exceed 4 pCi/L (picocuries per litre), equivalent to 148 Bq/m^3^^42^. The World Health’s Organization (WHO) guideline of 100 Bq/m^3^ is even more protective^7^, but notably, both the US EPA and WHO guidelines do not specifically include workplaces. The European Union has required its members to set a radon guideline of 300 Bq/m^3^ for dwellings and public buildings, including workplaces^43^. Health Canada’s radon guideline (200 Bq/m^3^ for dwellings and workplaces (not regulated by other radiation regulations)^10^, recognizes the ubiquity of radon gas^44^ in buildings but does not adequately protect workers exposed to levels below 200 Bq/m^3^. The American Conference of Governmental Industrial Hygienists (ACGIH) Threshold Limit Values (TLVs) for radon is an average annual effective dose of 20 mSv (milliseverts)^45^, and ACGIH TLVs have been adopted as guidelines or legal limits in many Canadian provinces^46^. Calculating an effective dose (mSv) (effective dose = radon level × time × dose coefficient^47^) requires making various assumptions that are beyond the scope of this discussion. Following assumptions defined by the International Commission on Radiological Protection (ICRP) (i.e., 2000 h; 6.7 × 10^–6^ mSv per Bq h/m^3^), an individual would receive an effective dose of 20 mSv (ACGIH TLV) from working in a building with a radon concentration of approximately 1500 Bq/m^3^^47,48^. Notably, the regulations based on this ACGIH TLV allows for radon levels that are significantly greater than the Health Canada guideline of 200 Bq/m^3^ for workplaces^10^.

The focus of this study was the prevalence and level of occupational exposure to radon, but also it also should be acknowledged that there is a significant contribution of environmental radon exposures to an individuals’ cumulative exposure over a lifetime. Recent studies have revealed increased our knowledge on environmental radon exposures in particular, including knowledge, risk perceptions, and homeowners’ testing habits^49–53^. A recent study by Khan et al. demonstrated that radon concentrations in Canadian residences have increased over time and modelling suggests that by 2050 the average residential level will reach 176 Bq/m^3^^54^. The increase is thought to be related to modern building construction and design practices which trap radon indoors^54^. Workplaces were not included in the predictive modelling, but changing building practices could presumably also have an impact in occupational indoor environments.

Occupational exposure to radon has not been characterized to the same extent as environmental exposures, with the exception of mining-related radon exposures^7,24,26,27^. Few other countries have estimated the prevalence of occupational radon exposure for their workforce. CAREX EU previously estimated that 2.7 million workers are exposed to radon in the European Union (based on employment data from the early 1990s), with Germany (820,000 workers exposed), Great Britain (560,000), and France (520,000) being the top 3 countries for occupational radon exposure^55^. TICAREX, the Costa Rican adaptation of CAREX, estimated that 13,800 workers are exposed to radon^56^. However, neither the CAREX EU nor TICAREX radon estimates were based on methods using measured exposures. Rather, the estimates were based on expert-based assessments that may have missed lower exposed workers, and thus likely underestimate the number of exposed workers. Radon surveys have been carried out in workplaces but have been generally limited^24^ to specific types of workplaces (e.g., schools/daycares, hospitals, government buildings)^18,51,57–61^. More radon surveys across a variety of industries and occupations with exposed workers, as outlined in this study, would help better characterize occupational exposure. Further inquiry is needed to better understand how homeowners’ knowledge, attitudes, and beliefs translate from environmental to occupational radon exposures. Studies could also explore the impact of modern building construction and design practices on increased radon concentrations in Canadian workplaces to see if greater exposure is to be expected outside of the residence. In looking to the future of radon exposure, the contribution of occupational versus residential radon exposure has shifted due to the COVID-19 pandemic and stay-at-home orders, and working from home is likely to remain a common practice in many industries, as nearly 40% of Canadians have jobs that can be done from home^62^.

There are several limitations to this study that are important to note. We had to rely on radon exposure measurements that were taken by Health Canada beginning in 2007, and some of these buildings may have been mitigated since if they exceeded the Health Canada guideline, which may have led to an overestimation of 2016 exposure levels in some cases. Additionally, only federal buildings were sampled in this campaign and this does not represent the wide variety of building types with different ownership (e.g., provincial or municipal buildings, private companies, people working out of homes, etc.). It is not clear how this would impact our estimates of exposure levels since it is likely that many of those buildings would have had higher or lower radon levels than the federal buildings. As there is no repository of all workplaces (or even a random sample across industry types), Health Canada’s federal buildings survey was the best available option.

There are also limitations to the CAREX approach that was used to both remove outdoor workers from the labour force to define the susceptible indoor working population, and the selection of exposure proportions for underground workers. These assessments were done on a consensus basis by three of the authors (CEP, CBG, PAD) who are all highly skilled occupational hygienists with decades of experience in occupational carcinogen exposure assessment.

The main strength of this study is that it describes the number of Canadians exposed to relatively low levels (> 100–200 Bq/m^3^) of radon in the workplace (e.g., general indoor workers) which is a novel contribution to the literature. Previous work by our team has demonstrated that due to the large size of this exposure group, it contributes the most to overall lung cancer burden from occupational radon exposure^39^. In the future, this work could also be used to update the estimates of the burden of lung cancer in Canada attributable to occupational radon exposure, which may be especially important given the changing nature of work locations and patterns due to the pandemic^38^. These estimates can be used by relevant stakeholders from across affected industries to support lung cancer prevention programs in all workplaces, not just ones considered traditionally at risk of exposure to radon.

This study presents the first attempt at assessing Canadian workers’ exposure to radon across all occupations and industries, including indoor workers. Overall, a significant number of workers are exposed to radon to levels exceeding 100 and 200 Bq/m^3^, and are thus not adequately protected by existing WHO or Canadian guidelines, respectively. The population level estimates can be strengthened through the systematic collection of radon measurements across multiple industries and occupations are needed to better characterize occupational exposure. Overall, study findings can be used to identify exposed workers, and to prioritize support lung cancer prevention programs within these groups.

## Methods

A two-pronged approach was used to estimate radon exposures among workers where direct contact to radon through uranium-containing materials is expected, and among indoor workers, both by province, sex, industry, and occupation. To estimate workers’ exposure through direct contact, the CAREX approach (described elsewhere^25^) was used. Briefly, occupations and industries at risk of exposure were identified using peer-reviewed and grey literature. Direct contact to radon was considered primarily among underground workers at risk of high exposure to radon. The labour force values for the identified occupation and industry intersections were obtained from the 2016 Canadian census^63^ by sex, province, industry (2012 North American Industry Classification System (NAICS), at the four digit level)^64^, and occupation (2016 National Occupation Classification (NOC), at the four digit level)^65^. A percentage of workers exposed for each occupation and industry intersection was developed using existing data sources and expert assessment, and then was applied to the labour force values to obtain the prevalence estimate of underground workers exposed to radon by sex, province, occupation, and industry. All underground workers identified using these methods were assigned to an exposure category of > 800 Bq/m^3^.

**Figure 2:**
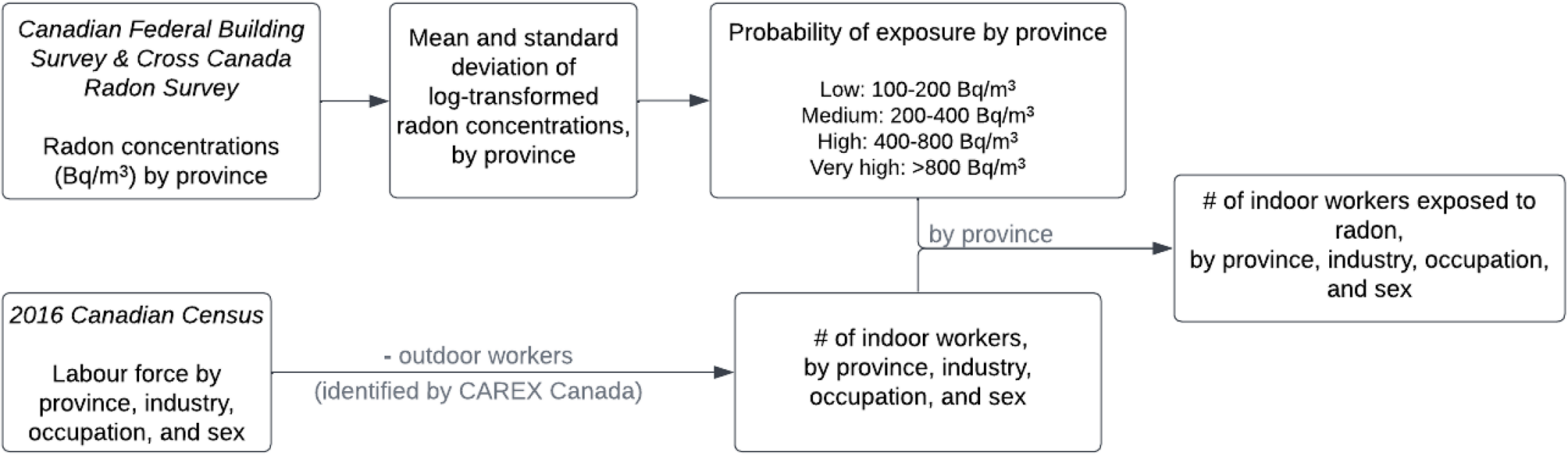
Flow chart of the methods used to estimate the number of indoor workers exposed to radon.

Data used to develop the estimates of indoor workers exposed to radon were collected from the Canadian Federal Building Survey and the Cross-Canada Survey of Radon Concentrations in Homes (CCRS)^13,66^. The Canadian Federal Building Survey, which has been conducted by Health Canada since 2007, measures radon concentrations in federal workplaces using alpha track detectors over at least 3 months. Since no federal buildings were surveyed in the region of Nunavut, the CCRS was used to calculate radon exposure for Nunavut only. The CCRS is a 2-year study conducted by Health Canada’s National Radon Program in 2009–2011. To estimate the number of indoor workers exposed to radon in various occupation and industry intersections, we modelled radon concentrations in workplaces and susceptible indoor working populations (Fig. 2). Indoor radon exposure was assessed for the working population reported in the 2016 Canadian census^63^, by sex, province, industry (2012 NAICS, at the four digit level)^64^, and occupation (2016 NOC, at the four digit level)^65^. The occupations and industries that employ indoor workers were identified by using the solar ultraviolet radiation estimates created by CAREX Canada to exclude outdoor workers^25^. Distributions of indoor workplace radon concentrations were calculated using the radon measurements from the two radon surveys, which were stratified by province. Lognormal distribution was used to model the indoor workplace radon exposure distributions. First, mean and standard deviation of log-transformed radon measurements from the surveys were calculated by province. These estimates were later used to calculate probabilities at log(100), log(200), log(400) and log(800) concentrations and for the ranges of interest. The distribution of radon concentrations was then applied to the susceptible (indoor worker) population model by province to determine the number of workers exposed. Occupation-, industry-, and province-specific exposures were estimated for four ranges of radon exposure that align with Canada’s NORM guidelines^10^ and the WHO recommended guideline for indoor residential spaces^7^; > 100–200 Bq/m^3^, > 200–400 Bq/m^3^, > 400–800 Bq/m^3^, and > 800 Bq/m^3^. As CAREX procedures use only publicly available, non-identifiable data, this research was deemed not to require research ethics review from Simon Fraser University.

## Data Availability

Radon measurement data can be accessed upon request from Health Canada (Radon Testing in Federal Building survey and Cross-Canada Survey of Radon Concentrations in Homes). CAREX Canada data are available online and upon request. Labour force data are available through Statistics Canada Census Program.
